# The Study of the Ability to Anticipate Future Events in Adolescents with Speech Disorders

**DOI:** 10.1192/j.eurpsy.2026.11256

**Published:** 2026-07-31

**Authors:** A. Akhmetzyanova, T. Artemyeva, A. Nauruzbaeva

**Affiliations:** 1 Department of Psychology and Pedagogy of Special Education, Kazan Federal University, Kazan, Russian Federation; 2 Nukus State Pedagogical Institute, Nukus, Uzbekistan

## Abstract

**Introduction:**

During adolescence, children become acutely aware of the necessity of planning their actions with regard to the anticipation of future events. Anticipation enables adolescents to shape their future, achieve higher academic success, manage interpersonal relationships, and adapt successfully within society. However, adolescents with speech disorders encounter difficulties in the process of regulating their behavior on the basis of subjective anticipation.

**Objectives:**

To examine the ability of adolescents with speech disorders to anticipate future events.

**Methods:**

The empirical study involved 94 adolescents aged 11 to 14 years (M = 12.46; SD = 0.659): 46 adolescents with speech disorders and 48 adolescents without speech impairments. The following instruments were employed in the study: *Anticipatory Competence Test* (V. D. Mendelevich); the method *Anticipation of the Outcome of a Situation Involving a Norm Violation* (V. P. Ulyanova); the questionnaire *Study of Anticipatory Competence in Adolescents* (A. I. Akhmetzyanova, T. V. Artemyeva); as well as statistical methods for data analysis (SPSS v.24).

**Results:**

It was found that adolescents with speech disorders experience difficulties in anticipating future events, encounter problems in controlling their own movements, orienting themselves in space, and planning time: the mean score for the indicator ‘general anticipatory competence’ does not reach the normative threshold (M = 238.67; Gn = 241). Adolescents with speech disorders were fairly successful in identifying external features of situations involving a violation of social norms: they understood when a norm was being violated, who the violator was, and who the victim was in the given situation. However, they experienced difficulties in responding to questions about the motives of other participants’ behavior (M = 8.91; Max = 32). A comparative analysis of anticipatory competence in adolescents with and without speech disorders was conducted using Student’s t-test, which revealed statistically significant differences: adolescents with speech disorders were less able to anticipate communicative situations, encountered difficulties in interactions with adults and peers, and struggled to formulate verbal utterances (T = 2.90); they had difficulties organizing their free time and were unable to plan their time adequately (T = 3.38); and they experienced difficulties in anticipating their parents’ verbal utterances and in communicating freely with their parents (T = 2.99).

**Conclusions:**

The results obtained in the study make it possible to develop programs aimed at enhancing the ability of adolescents with speech disorders to anticipate future events. This work was carried out with the financial support of the Russian Science Foundation under the project ‘Prognostic Competence of Adolescents with Disabilities: A Structural-Functional Model and the Potential of Digital Socialization’.

**Disclosure of Interest:**

None Declared

